# Screening of adult dental patients visiting Khyber College of Dentistry, Peshawar for HBV and HCV infections and identifying the associated risk factors

**DOI:** 10.12669/pjms.333.12260

**Published:** 2017

**Authors:** Jamila Haider, Ghosia Lufullah, Rubina Nazli, Tasleem Akhtar, Asma Shah

**Affiliations:** 1 Jamila Haider, BS, PhD Scholar. Lecturer, Centre for Biotechnology & Microbiology, University of Swat, Swat, Pakistan; 2 Prof. Dr. Ghosia Lutfullah, M.Phil, Ph.D. Director, Centre of Biotechnology & Microbiology, University of Peshawar, Peshawar, Pakistan; 3 Prof. Dr. Rubina Nazli, MBBS, PhD. Institute of Basic Medical Sciences Khyber Medical University, Peshawar, Pakistan; 4 Dr. Tasleem Akhtar, M. Phil, Ph.D. Senior Scientific Officer (Ex), PMRC Research Centre, Khyber Medical College, Peshawar, Pakistan; 5 Asma Shah, BS, PhD Scholar. Centre of Biotechnology & Microbiology, University of Peshawar, Peshawar, Pakistan

**Keywords:** Viral Hepatitis, Prevalence, Risk factors, Dental patients

## Abstract

**Objective:**

To screen out adult patients for HBV and HCV infections visiting Khyber College of Dentistry Peshawar (KCD) for different dental treatments and to identify the associated risk factors.

**Method:**

This cross-sectional study was conducted at Khyber College of Dentistry, Peshawar in the year 2013. A total of 1540 patients >15 years, visiting KCD for seeking different dental treatments were screened for hepatitis B & C. Informed consent was taken before blood collection and filling of a structured questionnaire. Blood samples were tested against HBsAg and anti HCV by using ICT that were further confirmed by ELISA. The data was analyzed using Epi info version 6.

**Results:**

A total of 1540 patients were screened during the study. Among these 36.4%(561) were males and 63.6%(979) were females. Overall prevalence of HBV and HCV was 5.12%(79). On screening, 2.14%(33) were found to be HBs Ag positive of which 2.5%(14) were males and 1.9%(19) were females. HCV was found positive in 2.98%(46) individuals having male to female ratio of 1.6% and 3.8%. Frequency of HBsAg was high in age group 56-65 year and HCV in 36-45 year group. Previous history of IV/IM injections, spouse patient of hepatitis, blood transfusion, surgical operation were found significant risk factors in the transmission of both hepatitis B and C, while previous history of dental treatment and sharing of clippers were significant risk factor in spreading hepatitis C infection only.

**Conclusions:**

Overall prevalence of HCV was higher than HBV. Previous history of injections, spouse patient of hepatitis, blood transfusion, surgical operation were found significant risk factors in the transmission of both hepatitis B and C.

## INTRODUCTION

Hepatitis is an inflammatory condition of the liver, most commonly caused by a viral infection. Of these viruses, hepatitis B virus (HBV) and hepatitis C virus (HCV) infections account for a substantial proportion of liver diseases worldwide.^1^ They are the major causes of severe liver disease, including hepatocellular carcinoma and cirrhosis-related end-stage liver disease.

Both HBV and HCV are blood-borne^2^ viruses with distinct routes of transmission. Modes of infection^3^ are more or less similar. HBV can be prevented with vaccine which is not possible in case of HCV.

Globally, Hepatitis B has been found to infect about 350 million people^4^ and result in 563 000 deaths annually. Most commonly, HBV infection is acquired by vertical transmission from an HBsAg positive mother or via horizontal transmission in childhood^5^ through blood and blood products and unsafe sexual contacts, intrafamilial transmission is also reported.^6^

Globally, hepatitis C virus (HCV) has infected an estimated 130 million people, most are chronically infected. HCV-infected people act as a reservoir for transmission to others and are at risk for developing chronic liver disease, cirrhosis, and primary hepatocellular carcinoma (HCC). Worldwide, HCV accounts for 27% of cirrhosis and 25% of HCC worldwide.^7^ The major modes^8^,^9^ of HCV transmission are use of contaminated needles and instruments in medical practice, unsafe blood and blood product transfusion, intravenous drug use, face and armpit shaving with unsterilized instruments by barbers, ear and nose piercing, poor personal hygiene habits and treatment (practice by non-qualified people). In Pakistan,^10^ the single most important cause of HCV transmission is lack of proper screening of the transfusion blood.

Pakistan^11^,^12^ is among the worst afflicted nations due its large population (165 million) and intermediate to high rates of infection. Estimated prevalence of chronic carrier state of Hepatitis B amongst high-risk groups in Pakistan^13^ ranges from 6-12% whereas prevalence of Hepatitis C in the high-risk population is much higher - ranging from 15-25%. More ever, it has also been estimated that in general population chronic carriers of Hepatitis C and Hepatitis B is 5% and 3% respectively.

These viruses are present in the saliva^14^ or blood of an infected patient therefore dentists and dental health care workers are at a high risk of acquiring infection. In Pakistan^15^-^18^, several previously conducted studies have shown different prevalence rates of HBV and HCV infection in general population. Many studies are also conducted on prevalence of HBV and HCV in dental patients, but no data is available on finding the risk factors for HBV and HCV in dental patient. Therefore the present study was designed to screen out adult patients for HBV and HCV infections, visiting KCD for different dental treatments and to identify the associated risk factors.

## METHODS

The present study was conducted at Khyber College of Dentistry Peshawar during the year 2013. Outdoor patients, referred from different sections of KCD, aged > 15 years and of both sexes were included in the study. The patients had different dental problems and visited for the treatments like scaling, dental extraction, dental filling, root canal treatment, minor oral/maxillofacial surgery. A total of 1540 participants were included in the study. Institutional ethical approval was taken and informed consent was taken from every patient. In patients age 16-17 years consent was taken from the parents/guardians. Brief personal, family and medical history was taken on a structured performa and data was analyzed using Epi info version 6.

The blood samples of all these patients were taken in the KCD lab under strict aseptic conditions by a qualified technician. Gel tubes were used for collection of blood samples (5ml). Sera were collected from these samples and were screened for HBsAg and Anti HCV using immunochromatography technique (ICT) in the PMRC (Pakistan Medical Research Council) labs of Khyber Medical College Peshawar. Positive tests were confirmed by ELISA method.

Test results were kept strictly confidential and were only conveyed to the participants. Those with a positive test result were given advice for further testing and treatment, and were referred to the nearest government health facility.

## RESULTS

A total of 1540 patients were screened during the study. Among these 561(36.4%) were males and 979(63.6%) females. The percentage of married and educated participants was 1265(82.1%) and 361(23.4%) respectively. On screening, 33(2.14%) were found to be HBs Ag positive of which 14(2.5%) were males and 19(1.9%) were females. HCV was found positive in 46(2.98%) individuals. Male and female distribution was 1.6% and 3.8% respectively. Overall percentage of HBV and HCV was 79(5.12%) as shown in Table-I.

**Table-I:** Overall Prevalence of HBV & HCV in patients of KCD Peshawar.

*Variables*	*N(1540)*	*%*
Male	561	36.4
Female	979	63.6
Married	1265	82.1
Educated	361	23.4
HBsAg Positive	33	2.14
Male	14	2.5
Female	19	1.9
HCV Positive	46	2.98
Male	9	1.6
Female	37	3.8
HBV & HCV Positive	79	5.12

An age-wise distribution of infected patients is shown in Table-II. HBs ratio was high in age group 56-65year and HCV in 36-45 years group. Different risk factors associated with prevalence of HBV and HCV are shown in Table-III.

**Table-II:** Age wise distribution of patients visiting KCD for dental treatment.

*Age Group(Years)*	*N*	*%*	*HBsAg Positive*		*HCV Positive*	

			*N*	*%*	*N*	*%*
16-25	358	23.2	3	0.8	6	1.7
26-35	549	35.6	13	2.4	15	2.7
36-45	339	22.0	9	2.7	18	5.3
46-55	176	11.4	5	2.8	3	1.7
56-65	97	6.3	3	3.1	3	3.1
>66	21	1.4	0	0	1	4.8

Total	1540	100	33	2.14	46	2.98

**Table-III:** Risk factors associated with the prevalence of HBV and HCV in dental patients.

Risk Factor	*Patients (1540)*		*Hepatitis B Positive(N=33)*				*Hepatitis C Positive (N=46)*			
	*N*	*%*	*N*	*%*	*OR(95% CI)*	*P*	*N*	*%*	*OR(95% CI)*	*P*
Male	561	36.4	14	2.5	1.45(0.63-2.61)	0.47	9	1.6	0.41(0.19-0.87)	0.01
Female	979	63.6	19	1.9			37	3.8		
Married	1265	82.1	29	2.3	1.59(0.55-4.56)	0.38	43	3.4	3.19(0.98-10.36)	0.14
Unmarried	275	17.9	4	1.5			3	1.1		
Educated	361	23.4	8	2.2	1.05(0.47-2.34)	0.91	9	2.5	0.79(0.37-1.66)	0.54
Un-Educated	1179	76.6	25	2.1			37	3.1		
***Spouse patient of Hep B / Hep C***										
Yes	25	2	2	8	4.60(1.02-20.67)	0.02	7	28	9.64(3.92-23.75)	0.0001
No	1240	98	23	1.9			36	2.9		
***Hx jaundice Self***										
Yes	519	33.7	15	2.9	1.67(0.83-3.32)	0.14	15	2.9	0.95(0.51-1.77)	0.87
No	1021	66.3	18	1.8			31	3.0		
***Hx. Injections***										
Yes	351	22.8	13	3.7	2.25(1.11-4.57)	0.02	11	3.1	2.69(1.34-5.41)	0.003
No	1189	77.2	20	1.7			35	2.9		
***Hx. Blood Transfusion***										
Yes	35	2.3	4	11.5	6.57(2.18-19.81)	0.001	5	14.3	5.91(2.18-16.01)	0.0007
No	1505	97.7	29	2.1			41	2.7		
***Surgical Operation***										
Yes	167	10.8	9	5.4	3.20(1.46-7.01)	0.002	11	6.5	2.69(1.34-5.41)	0.003
No	1373	89.2	24	1.7			35	2.5		
***Previous Dental treatment***										
Yes	274	17.8	9	3.3	1.76(0.81-3.82)	0.15	14	5.4	2.08(1.09-3.94)	0.02
No	1266	82.2	24	1.9			32	2.5		
***Sharing of clippers***										
Yes	1517	98.5	33	2.2	-	0.47	42	2.7	0.13(0.04-0.41)	0.0004
No	23	1.5	0	0			4	17.4		
***Sharing of tooth brush***										
Yes	27	1.7	2	7.4	3.82(0.87-16.86)	0.05	1	3.7	1.25(0.16-9.45)	
No	1513	98.3	31	2.04			45	2.9		0.82
***Trimmer/Blade***										
Yes	434	77.5	9	2.1	0.87(0.23-3.26)	0.83	8	1.8	2.30(0.28-18.6)	0.42
No	126	22.5	3	2.4			1	0.8		
***Visit to Barber Shop***										
Yes	525	93.8	9	1.7	0.19(0.04-0.72)	0.03	9	1.7	-	0.43
No	35	6.2	3	8.6			0	0		
***Ear/Nose pierced***										
Yes	877	89.5	17	1.9	0.38(0.13-1.07)	0.05	31	4.2	1.22(0.37-4.07)	0.74
No	103	10.5	5	4.9			3	1.1		
***Haemodialysis***					-					
Yes	6	0.4	0	0		0.71	1	16.7	6.62(0.75-57.8)	0.04
No	1534	99.6	33	2.2			45	2.9		
***Acupuncture***					-					
Yes	9	0.6	0	0		0.65	0	0	-	0.59
No	1531	99.4	33	2.2			46	3.0		

### Spouse Patients History

In case of spouse patient 2(8%) were positive for HBsAg [OR 4.601;CI 1.02-20.67], while anti-HCV was positive in 7(28%) [OR 9.64; CI 3.92-23.75] with significant value *p*< 0.02 and *p<*0.0001 respectively.

### Therapeutic injections

Results shows that 13(3.7%) dental patients having a history of therapeutic injections were positive for HBsAg having *p <*0.02 (Table-III). Anti-HCV antibody was positive in 11(3.1%) cases with previous history of therapeutics injections [OR 2.69; CI 1.35-5.41].

### History of blood transfusion and surgical operation

HBsAg was reported as positive in 4(11.5%) patients having a history of blood transfusion with statically significant value *p<*0.001. On other hand 5(14.3%) cases were positive for anti-HCV antibody in previously blood transfused patients with a significant value of *p<*0.0007.9(5.4%) cases were positive for HBsAg having a history of surgical operation. Anti-HCV antibody was positive in 11(6.5%) cases with a history of surgical operation having a significant value *p<*0.002 and p<0.003 respectively.

### Dental treatment

Among 274 (17.8%) patients with a previous history of dental treatment like extraction, scaling and filling, 14(5.4%) were HBsAg positive having a statistically significant value *p<*0.02, while it was not a significant risk factor in case of HCV.

### Haemodialysis

There was only one case reported as HBsAg positive in dental patients with a previous history of haemodialysis.

### Sharing of tooth brush/clippers

These were significant risk factors in case of HBV and HCV with statistically significant value *p*<0.0004 and *p<*0.05 respectively.

### Visit to barber/piercing ear & nose

In case of HBsAg positive cases, 9 (1.7%) visited barber shop [OR 0.19; CI 0.04-0.72] and 17(1.9%) were having experience of piercing ear & nose with significant value *p*< 0.05.

## DISCUSSION

The overall prevalence ofof HBV and HCV in this study was 79(5.12%). On screening, 33(2.14%) were found to be HBs Ag positive while HCV positive accounted for 46(2.98%). Viral Hepatitis is a major problem throughout the world. There have been studies regarding the prevalence of hepatitis B surface antigen (HBsAg) and anti-hepatitis C antibody (HCVab). However, the majority of these have reported a variety of rates, depending on their study population, which limits the generalizability of their results to the general population. On the other hand, cultural diversity in the different cities of Pakistan also necessitates the performing separate population-based studies in the various regions.

A review study conducted by Huma et al^19^ on prevalence and risk factors of HBV and HCV in general population of Pakistan and showed an overall prevalence in adults 2.4% for HBV and 3.0% for HCV. Another study conducted by Javed et al^20^ reported an overall prevalence of 2.8% for HBV and 3.19% for HCV in general population of NWFP.

A similar study conducted on the dental patients visiting Bacha Khan Medical & Dental College^21^ showed a prevalence of 7.75% for HCV and 7.0% HBV with no significant difference in males and females. This ratio is quite higher than we found in our study. However, there is statistical significance among gender based prevalence in HBV as well HCV.

Another study conducted at dental sector of Ayub Medical College Abbottabad^22^, showed total infection with HBV & HCV as 4.1% which is quite lower than our findings. The percentage of female was higher that correlates with our findings. However, in above mention studies HCV is more prevalent than HBV.

Another study conducted by Dilhan et al in Istanbul^23^ on dental patients showed almost similar prevalence of HBV i.e. 2.3% but a lower prevalence of HCV i.e. 0.1%. However, males were more prone to be infected in contrast to our study.

According to another study conducted at KCD^24^ in 2005, the percentage of HBV was 1.66% and HCV was 1.26%. The present study reported higher prevalence that indicates an increase in the incidence rate of HBV & HCV in our region.

The major risk factors for HBV and HCV in general population, as reported by Huma et al^19^ are contaminated needle use in medical care, IDUs, unsafe blood and blood transfusion, shaving by barbers and spousal transmission. Another similar study conducted by Ahmed et al^25^ reported reuse of syringes, shaving by barbers, sharing of smoking utensils, ear / nose piercing as the risk factors for HBV and HCV. Additionally, tattooing was reported as risk factor for HCV.

A review conducted by Nima et al^26^ on the dental treatment as a risk factor for HBV and HCV showed that although weak, there is an all-time risk of HBV and HCV infection during dental treatment. The risk factors reported by our study are same as reported by these authors in general population with additional factor of history of surgical operation and sharing of tooth brush as risk factor for HCV only.

### Limitation of Study

The limitations of this study are small sample size and collection of samples only from one dental hospital. Work must be conducted and data should be collected from different dental settings.


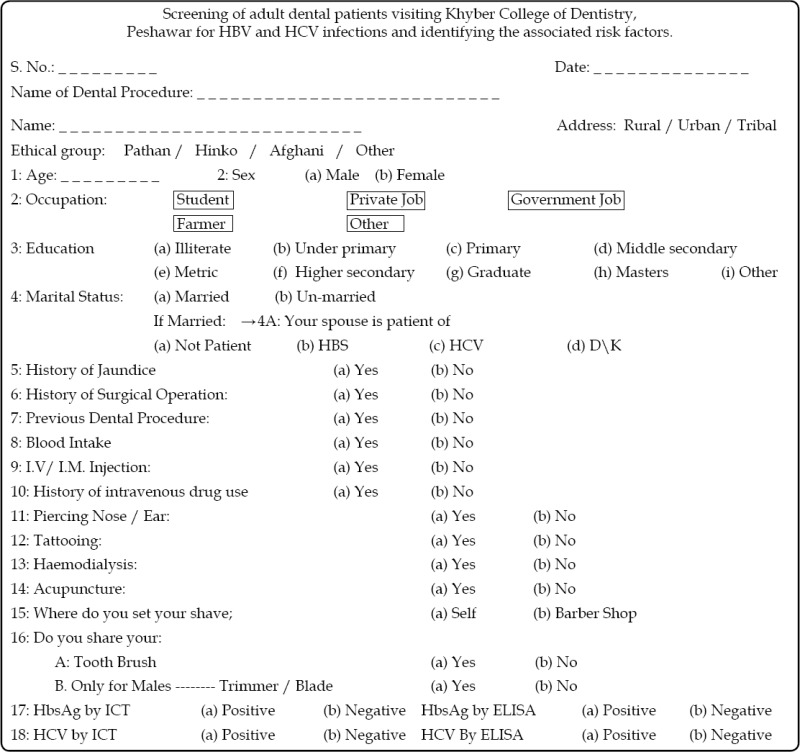


## CONCLUSION

Prevalence of anti-HCV antibody and HBsAg is more common in our study. Previous history of injection, blood transfusion, surgical operation, sharing of tooth brush/clippers, visiting barber shop, nose/ear piercing and dental treatment/surgery were observed as risk factors for transmission of anti-HCV antibody and HBsAg in dental patients.

### Authors’ Contribution

**JH, RN** conceived, designed and did statistical analysis & editing of manuscript.

**GL, JH, AS** did data collection and manuscript writing.

**TA** did review and final approval of manuscript.
